# Genomic investigation of the emergence of *vanD* vancomycin-resistant *Enterococcus faecium*

**DOI:** 10.1099/acmi.0.000712.v3

**Published:** 2023-12-04

**Authors:** Sarah L. Baines, Romain Guérillot, Susan Ballard, Paul D. R. Johnson, Timothy P. Stinear, Sally Roberts, Benjamin P. Howden

**Affiliations:** ^1^​ Department of Microbiology and Immunology, The University of Melbourne at the Peter Doherty Institute for Infection and Immunity, Melbourne, Victoria, Australia; ^2^​ Microbiological Diagnostic Unit Public Health Laboratory, Department of Microbiology and Immunology, The University of Melbourne at the Peter Doherty Institute for Infection and Immunity, Melbourne, Victoria, Australia; ^3^​ Department of Infectious Diseases, Austin Health, Melbourne, Victoria, Australia; ^4^​ Department of Microbiology, LabPlus, Auckland City Hospital, Auckland, New Zealand

**Keywords:** *Enterococcus*, vancomycin resistance, *vanD*, comparative genomics, integrative conjugative elements

## Abstract

Vancomycin-resistant *Enterococcus* (VRE) is an increasingly identified cause of human disease, with most infections resulting from the *vanA* and *vanB* genotypes; less is known about other clinically relevant genotypes. Here we report a genomic exploration of a *vanD* VRE *faecium* (VREfm), which arose *de novo* during a single infectious episode. The genomes of the vancomycin-susceptible *E. faecium* (VSEfm) recipient and resulting VREfm were subjected to long-read sequencing and closed, with whole-genome alignments, cross-mapping and orthologue clustering used to identify genomic variation. Three key differences were identified. (i) The VREfm chromosome gained a 142.6 kb integrative conjugative element (ICE) harbouring the *vanD* locus. (ii) The native ligase (*ddl*) was disrupted by an IS*Efm1* insertion. (iii) A large 1.74 Mb chromosomal inversion of unknown consequence occurred. Alignment and phylogenetic-based comparisons of the VREfm with a global collection of *vanD*-harbouring genomes identified strong similarities in the 120–160 kb genomic region surrounding *vanD,* suggestive of a common mobile element and integration site, irrespective of the diverse taxonomic, geographical and host origins of the isolates. This isolate diversity revealed that this putative ICE (and its source) is globally disseminated and is capable of being acquired by different genera. Although the incidence of *vanD* VREfm is low, understanding its emergence and potential for spread is crucial for the ongoing efforts to reduce antimicrobial resistance.

## Data Summary

All sequence data generated in this study have been uploaded to the European Nucleotide Archive under BioProject PRJEB42923.

**Impact Statement**

Vancomycin-resistant enterococci are a pathogen of increasing concern, with *vanD* representing a rare but clinically important VRE genotype. The limited number of reported cases suggests an evolutionary pattern of *de novo* emergence; the *vanD* locus thought to be acquired from the patient’s own gut microbiome during prolonged and/or complicated antimicrobial therapy. Here we report the *de novo* emergence of a *vanD* VREfm, demonstrating through comparative genomic analysis of paired isolates the likely evolutionary steps taken to become VRE. Further, we place this isolate in the context of publicly available *vanD*-harbouring sequences and provide a current, summative description of the mechanism of *vanD* VRE development.

## Introduction

The genus *Enterococcus* comprises human pathogenic bacteria associated with a diverse range of disease manifestations and high mortality when nosocomially acquired [1, 2]. The emergence of antibiotic resistance is a further concern, with vancomycin-resistant *Enterococcus* (VRE) listed by the World Health Organization as a high-priority target for the development of novel antimicrobials and therapeutic approaches [3]. The large majority of human enterococcal disease is caused by *Enterococcus faecalis* and *Enterococcus faecium *; vancomycin resistance in these lineages is predominantly represented by the *vanA* or *vanB* genotypes [4]. There are, however, an increasing number of alternative *van* genotypes harboured by enterococci and other bacterial species, many linked with clinical disease: *vanC*, *vanD*, *vanE*, *vanG*, *vanL*, *vanM* and *vanN* [4–6].

First described in 1997 [7], *vanD* is a clinically relevant but uncommonly identified *van* genotype. The ~5.7 kb *vanD* locus encodes six proteins: a dehydrogenase (*vanH*), a ligase (*vanD*), a dd-dipeptidase (*vanX*), dd-carboxypeptidase (*vanY*) and a two-component regulatory system (*vanR*, *vanS*) [8]. A seventh gene (*intD*) has been inconsistently identified, encoding a putative integrase-like protein [9, 10]. Five *vanD* subtypes have been described based on the amino acid identity of *vanD* [8, 11–15]. With the exception of subtype vanD2, the *vanD* locus is constitutively expressed [15]; almost all genetically characterized *vanD* VRE contain *vanR* and/or *vanS* mutations, and/or an impaired or disrupted native ligase (encoded by *ddl*) [9, 16].

A few studies have reported on related genomic islands (GIs) associated with the *vanD* locus [11, 16, 17]; carried in different genera and divergent lineages of enterococci. The genes present in this region are suggestive of an integrative conjugative element (ICE). However, the region, while able to be circularized, has not been shown to be mobilizable, and no source for the *vanD* locus or putative ICE has been conclusively identified [9–11, 13–16, 18, 19], although a recent study has demonstrated that the ICE encompasses genes consistent with those from mobile elements recovered in various anaerobic genera belonging to the order Eubacteriales [17]. The limited number and geographical separation of reported *vanD* VRE suggest that these isolates have arisen primarily through *de novo* acquisition of the *vanD* locus [16]. This is a concern, as there remains a poor understanding of this phenomena and, importantly, what factors promote this horizontal gene transfer.

Here we report a genomic investigation into the emergence of a *vanD* vancomycin-resistant *E. faecium* (VREfm) in New Zealand, which arose *de novo* during a complex patient infection. Reconstruction of the complete genomes of the vancomycin-susceptible *E. faecium* (VSEfm) recipient and resulting VREfm enabled characterization of the acquired *vanD* locus and the putative ICE in which it was carried. Comparison to publicly available *vanD*-harbouring sequences identified strong similarities between the *vanD* locus, putative ICE and integration site, suggestive of a common mechanism for horizontal transfer of *vanD* and a globally dispersed source for this ICE. Collectively, this works provides a summative description of our understanding of the dynamics surrounding the *de novo* development of *vanD* VRE.

## Methods

### Bacterial isolates

Isolates were recovered from primary clinical samples using standard laboratory procedures. The first isolate was a VSEfm (named AUS2001), recovered from a blood culture alongside a *Morganella morganii* and *Enterobacter cloacae *. The second isolate was a phenotypic VREfm (named AUS2002), recovered 68 days later from a hepatic abscess alongside a *Pseudomonas aeruginosa* and *Lactobacillus rhamnosus *. The minimum inhibitory concentrations (MICs), determined by the hospital labratory at the time of isolation using Etest, were 1 and ≥256 mg l^−1^ for vancomycin, and 2 and ≥256 mg l^−1^ for teicoplanin, for the VSEfm and VREfm, respectively.

### Whole-genome sequencing and genome assembly

Both AUS2001 and AUS2002 underwent DNA extraction (Blood and Tissue kit, Qiagen), following the manufacture’s recommended protocol. Sequence libraries were prepared with the Nextera XT DNA Preparation kit (Illumina), and whole-genome sequencing was performed on an Illumina MiSeq with 2×300 bp paired-end chemistry. Genomic DNA was additionally sequenced on the PacBio RS-II with P4+C6 chemistry at the Duke Center for Genomic and Computation Biology (https://genome.duke.edu/).

Genome reconstruction was performed as described elsewhere [20]. Briefly, long-read sequences were assembled using the HGAP3 pipeline (SMRTPortal v2.3.0) [21]. Default parameters were used with the following exceptions: (i) seed read length was set to ‘1 kb’, (ii) minimum read quality was set to ‘0.80’, (iii) genome size was set to ‘3 000 000’ bp and (iv) the overlapper error rate was set to ‘0.04’. Following assembly, contigs were circularized with berokka v0.2.1 (github.com/tseemann/berokka), annotated with Prokka v1.13.3 [22] and reorientated to *dnaA* (chromosome) or *repA* (plasmid). All contigs were checked manually for assembly errors, as outlined elsewhere [20], and the final sequences were polished in an iterative manner with the short-read Illumina data using snippy v4.3.6 (github.com/tseemann/snippy), until no variants were detected. *In silico* MLST was performed using mlst v2.19.0 [23]. The complete genomes of AUS2001 and AUS2002 have been uploaded to the European Nucleotide Archive under BioProject PRJEB42923.

### Bioinformatic analysis

*De novo* assemblies of the short-read data were performed using SPAdes v.3.13.0 [24], excluding the pre-assembly error correction (‘--only-assembler’), including post-assembly error correction (‘--careful’) and a minimum coverage threshold of five (‘--cov-cutoff’) to remove potential contaminants. Mapping-based analyses were performed using snippy v4.6.0 (github.com/tseemann/snippy), with a minimum proportion for variant evidence (--minfrac) of 90 %. For cross-mapping of AUS2001 and AUS2002, the complete genomes and short-read *de novo* assemblies of each were used as a reference to map the short-read datasets against. Mutations identified in both cross-mapped alignments and not self-mapped alignments were enumerated to estimate the core genetic differences between the two isolates, as described elsewhere [25]. Whole-genome comparison of both isolates was performed using blast 2.2.26 [26], and visualized with Artemis Comparison Tool v17.0.1 [27]. Further, orthologue clustering to identify all shared and variable coding sequences was performed with roary v3.13.0 [28], using a nucleotide threshold of 99 % without splitting paralogues.

The publicly available *vanD*-harbouring sequences used in this study are listed in the Table S1, available in the online version of this article. For phylogenetic inference, alignment of the vanD protein, *vanD* locus (defined as the region from *vanR to vanX*) and the putative integrative conjugative element (defined as the region between the identified 13 bp direct repeats TTCCC[g/a/c]AC[a/g]ATGA) were generated and neighbour-joining phylogenetic trees built using ClustalW2 [29]. Trees were visualized in FigTree v1.4.3 (http://tree.bio.ed.ac.uk/software/figtree/). The vanD protein alignment was also used to categorize sequences by *vanD* subtype, using the previously reported subtype threshold of ≥97 % sequence identity [13].

## Results and discussion

### Comparison of AUS2001 (VSEfm) and AUS2002 (VREfm)

*E. faecium* AUS2001 (VSEfm) and AUS2002 (VREfm) represent paired isolates recovered from the same patient 2 months apart during a single, extended admission to a healthcare institution in New Zealand. The recovery of AUS2002 overlapped in time with a large nosocomial outbreak of *vanB* VREfm in the same institution. Subsequently, AUS2002 was referred to the country’s central public health laboratory for genotyping. Discordance between the vancomycin phenotype and *vanA/B* PCR performed instigated further testing of both AUS2001 and AUS2002. This revealed the isolates to be genetically similar by pulse field gel electrophoresis (PFGE) (Fig. S1), and AUS2002 to carry a *vanD* gene (by PCR). No other *vanD* VRE had been recovered in the country around this time, leading to the hypothesis of *de novo* acquisition, with transfer of the *vanD* locus suspected to have occurred from the patient’s own gut microbiome [30], driven by the extensive and complicated antimicrobial therapy given to the patient during their admission. This unusual finding promoted further genomic investigation to understand the development of AUS2002.

In confirmation of the original PCR and PFGE analysis performed in New Zealand, the genomes of AUS2001 (3 096 160 bp; accession OU015345–OU015349) and AUS2002 (3 241 449 bp; accession OU015350–OU015354) were found to be highly similar. Both genomes belonged to multilocus sequence type (ST) 203. Representing a globally disseminated, clade A enterococcal lineage, ST203 is also a major clone of hospital-associated VRE and has previously been identified to harbour *vanD* [6, 11]. AUS2001 and AUS2002 maintained the same four plasmids and cross-mapping of the genomes only identified two single-nucleotide variants, one single-nucleotide polymorphism and one single-base insertion (Table S3). Orthologue clustering of coding sequences in both genomes identified more variation. A total of 2892 clusters were identified using a nucleotide threshold of 99 %. Of these, 2718 clusters were conserved in both genomes and 222 were variably present (Tables S4–S8); 118 of the variable clusters represented the mobile element described below.

**Fig. 1.**
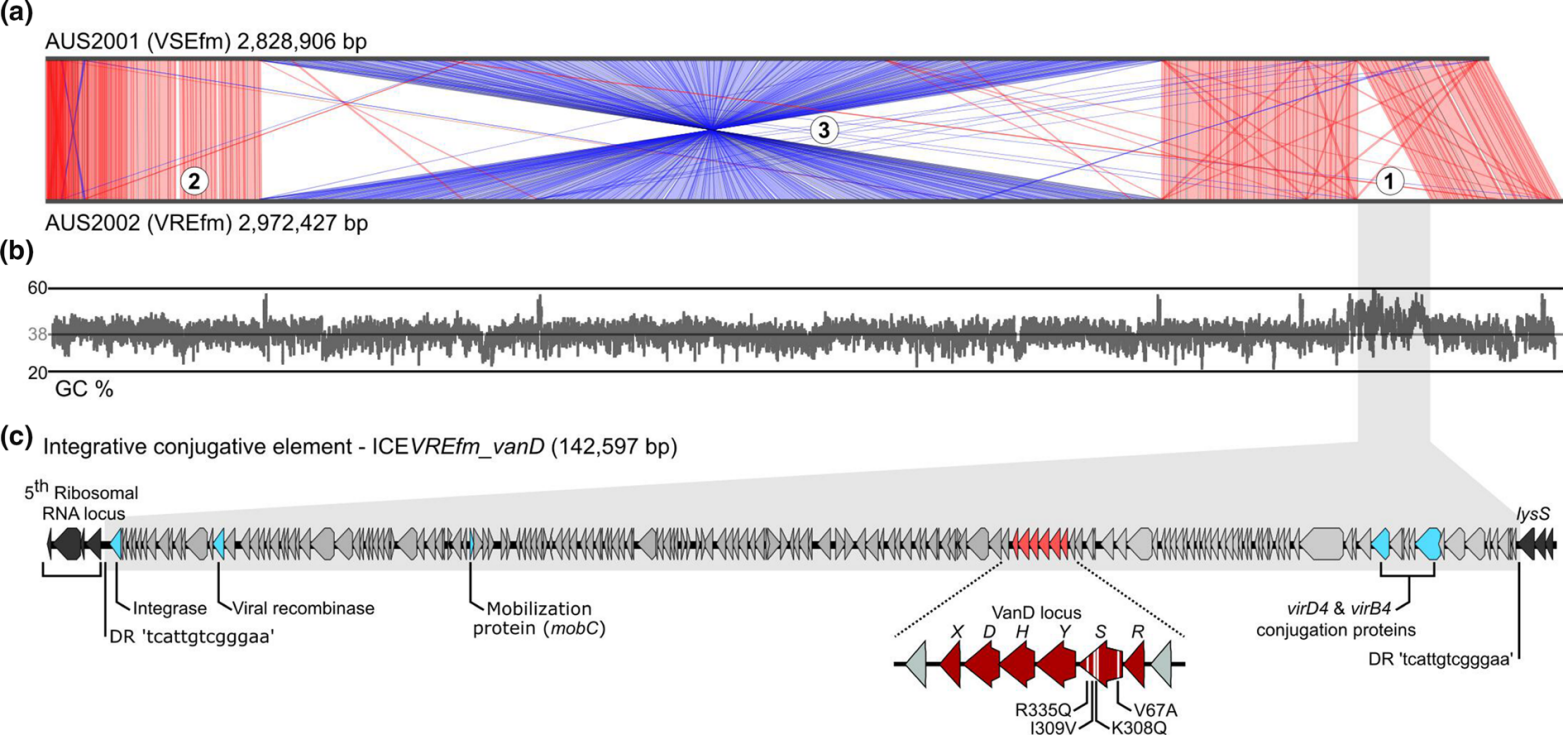
Genomic location and structure of the putative integrative conjugative element (ICE) harbouring the *vanD* locus. (**a**) Whole-genome alignment of AUS2001 (VSEfm) and AUS2002 (VREfm). Only blast matches of ≥90 % nucleotide identity are shown; red indicates matches in the same orientation and blue indicates opposite orientation. Highlighted are the three major structural changes identified: (1) integration of the putative ICE, (2) disruption of *ddl* through gain of an *IS*Efm1 and (3) a large chromosomal inversion. (**b**) Graph of the GC content across the genome of AUS2002, highlighting the increase in GC% in the ICE region. (**c**) Schematic of the ICE, highlighting the genes constituting an incomplete conjugation system and the *vanD* locus. Mutations identified in *vanS* are annotated and considered to mediate constitutive expression of the *vanD* locus.

Whole-genome alignment of AUS2001 and AUS2002 identified three large structural genomic differences, all within the chromosome (Fig. 1); two were considered the most relevant to the change in vancomycin susceptibility. First, AUS2002 contained an insertion of 142 597 bp, the region flanked by 13 bp direct repeats (5’ TCATTGTCGGGAA 3’) and encoding a putative 171 proteins (Fig. 1, Table S2). This included an integrase (*int*) gene and the three most conserved components of conjugative elements, a relaxase (*mobC*), a coupling protein (*virD4*) and the ubiquitous type IV secretion system (*virB4*) (Fig. 1c) [31]. Further, the region maintained a higher average GC content (44 %) than the rest of the genome (38 %) (Fig. 1b), strongly suggestive of horizontal gene transfer from a non-enterococcal host. Integration occurred directly downstream of the fifth (of six) ribosomal RNA loci disrupting *lysS* (Fig. 1c), as has been previously described [16]. The integrated region also contained the *vanD* locus – the source of the acquired vancomycin resistance in AUS2002. Based on these observations, this element was deemed a putative ICE that we have termed ICE*VREfm_vanD*. Of note, the mobility-associated genes identified encoded an incomplete system, notably missing an excisionase. While the ICE may encode an unidentified excision module such as a transposase [32, 33], it may also be the case that ICE*VREfm_vanD* is not fully autonomous and relies on an externally encoded trans-acting protein(s) or utilizes a different mechanism for transfer. The lack of evidence for excision of the ICE is consistent with the *vanD* VRE literature [10, 13–15, 17–19].

Second, the *vanD* genotype (excluding subtype vanD2) has invariably been associated with *vanRS* mutations and/or impairment/disruption of the native chromosomal ligase, encoded by *ddl* [9, 16, 34, 35]. Examination of the *ddl* gene in AUS2002 found an *IS*Efm1 inserted centrally in the coding region of the transposase at nucleotide position 759/1077, consistent with previous reports [12, 19]. It is predicted that this insertion will prevent production of the normal d-ala-d-ala peptide. While this would augment the efficiency of the resistance mechanism (maximizing inclusion of the new d-ala-d-lac peptide, which is unaffected by vancomycin [18], in the cell wall), it renders AUS2002 dependent on the acquired *vanD* ligase for cell wall cross-linking. As the sole functional ligase, loss of *vanD* through excision of the ICE would likely be highly detrimental if not lethal to the cell and may be promoting stabilization and maintenance of the ICE. AUS2002 can be grown both in the presence and absence of vancomycin, suggesting that the system is constitutively expressed in this isolate. Further, four mutations were identified in *vanS* (Fig. 1c); all (V67A, K308Q, I309V and R335Q) have previously been identified and linked to constitutive expression of the *vanD* locus [9].

Third, was a large chromosomal inversion of 1.74 Mb (Fig. 1a), occurring between two ribosomal RNA loci. It is unclear whether the inversion was prompted by the integration of ICE*VREfm_vanD* or has occurred independently. However, other large chromosomal inversion events have been reported to have coincided with *vanD* acquisition [16], suggestive of a potential, possibly transient, state of genomic instability surrounding the integration event. In the case of AUS2002, the region inverted does not overlap with the integration site of the ICE. It is unclear what phenotypic impact this inversion (or those reported in other isolates) has had, but it is an avenue for future investigations when exploring the phenomena of *de novo* acquisition of *vanD*.

### Comparison of AUS2002 with all available *vanD*-harbouring genomes

To place AUS2002 in the context of other *vanD*-harbouring genomes, a search of the *vanD* literature and the National Center for Biotechnology Information’s (NCBI’s) sequence databases was completed, with 49 sequence records representing unique isolates identified (Table S1).

**Fig. 2.**
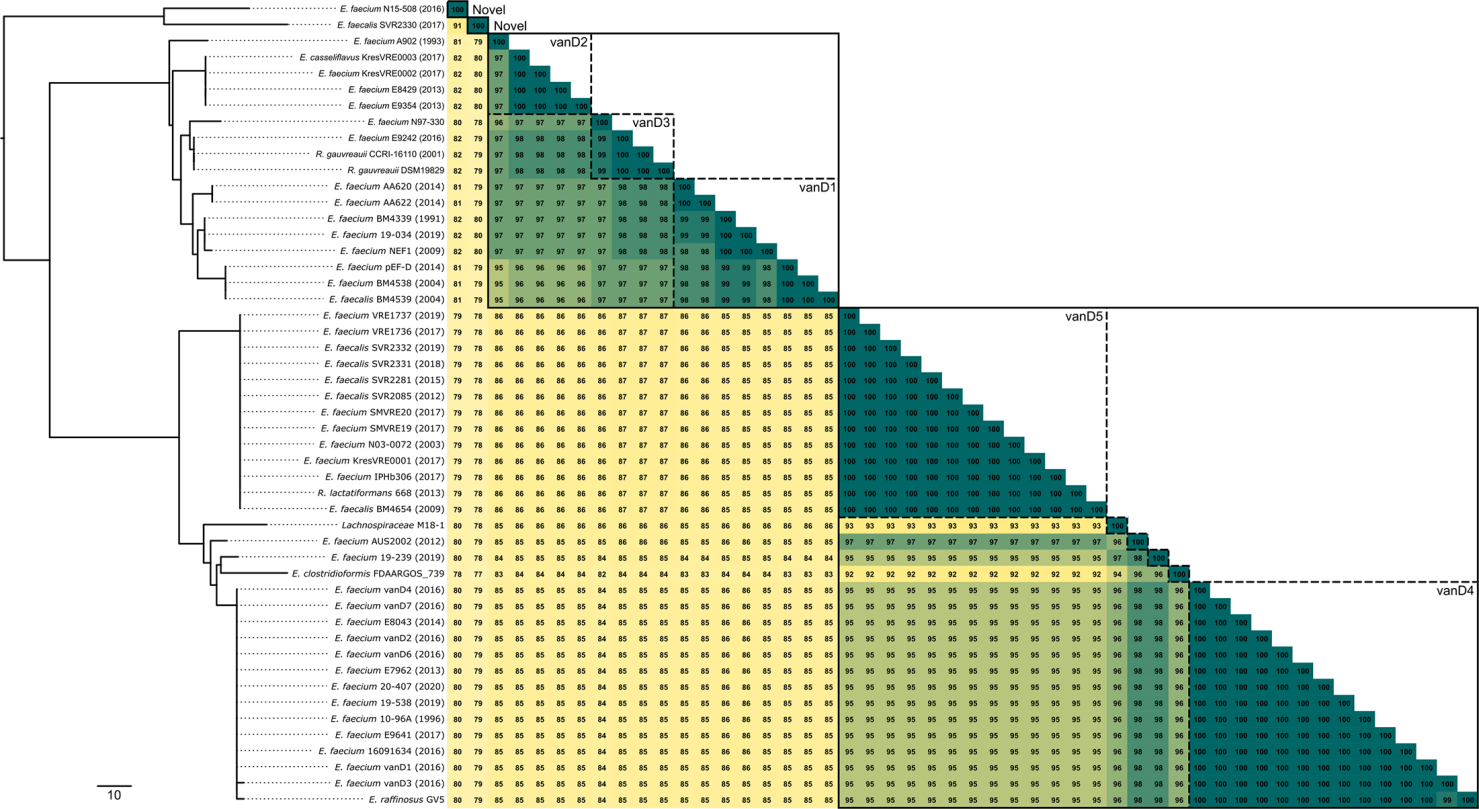
Diversity of the vanD protein sequence. A neighbour-joining phylogenetic tree was generated from an alignment of the vanD protein for all identified vanD-harbouring sequences (*n*=50) using ClustalW. The pairwise percentage amino acid identity is provided in the heatmap. Clusters have been defined using a 97 % identity threshold; solid lined boxes indicate inclusive groups (i.e. sharing ≥97 % identity with one other member) and dotted line boxes indicate explicit groups (i.e. sharing ≥97 % identity with all members). Scale bar represents number of sites.

Comparison of sequences from all 50 isolates identified that the vanD protein in AUS2002 is most closely related to the vanD4 subtype (Fig. 2), sharing 98 % amino acid identity with all other vanD4 protein sequences. However, AUS2002 also shared 97 % amino acid identity with all vanD5 protein sequences. Historically, the vanD subtypes have been defined with a 97 % amino acid identity threshold. But AUS2002 and other more recently identified vanD proteins have highlighted that this threshold no longer enables classification of vanD proteins into discrete subtypes, with vanD subtypes 1, 2 and 3 forming one phylogenetically congruent group, and subtypes 4 and 5 a second group alongside other related vanD proteins (Fig. 2). Two novel vanD protein subtypes were also identified and form a distinct monophyletic cluster in the vanD protein tree (Fig. 2).

**Fig. 3.**
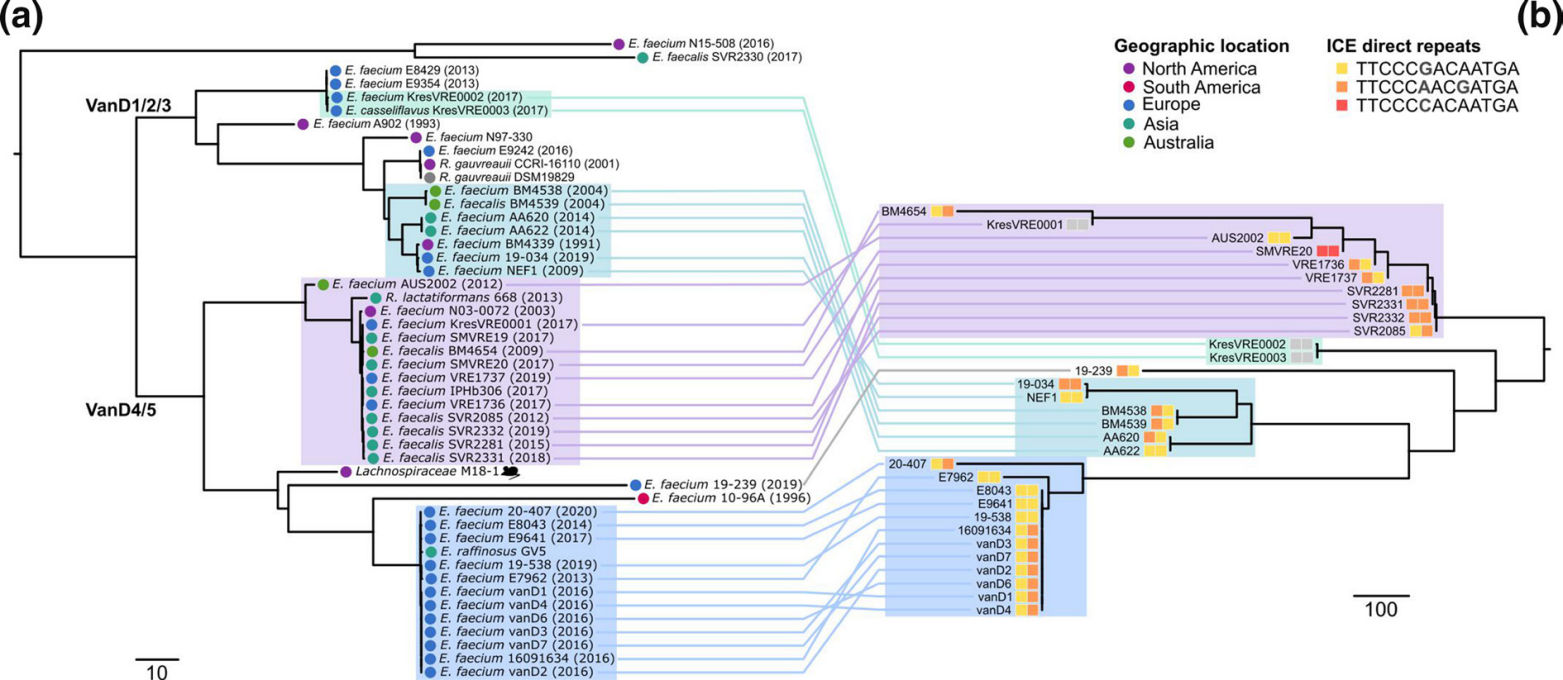
Phylogenetic congruence of the *vanD* locus and putative integrative conjugative element. Neighbour-joining phylogenetic trees were generated from alignments of the *vanD* locus (**a**), *n*=48 sequences, and the putative integrative conjugative elements (**b**), *n*=31 sequences, using ClustalW. Sequences represented in both trees are joined to highlight similarities in topology. Individual isolate information on taxonomy, geographical continent (tip circle colour) and year of recovery (value provided in parentheses of tip annotation), host (the single animal isolate indicated with a mouse symbol) and the direct repeat sequences (tip square colour) are provided in the tip labels and symbols (refer to key). Scale bar represents number of sites.

A phylogenetic tree constructed from an alignment of the full *vanD* locus for 48/50 isolates (2 sequence records did not contain the full locus sequence) (Fig. 3a) revealed a topology largely consistent with the tree constructed for the *vanD* peptide (Fig. 2). Of note, the topology did not show any strong correlation with the geographical location or year of isolation for isolates, or the species the sequence was recovered from. This finding is consistent with the hypothesis that *de novo* generation is the predominant mechanism by which *vanD* VRE emerge.

The putative ICE in AUS2002 was found to share significant similarity in sequence and structure to the 30 other full-length mobile elements identified in the *vanD* harbouring sequences (Fig. 3b). The integration site (in *lysS*) was conserved in all isolates (*n*=36 sequences with partial or full-length ICE), irrespective of the taxonomic, geographical and host origins of the isolates (Table S1). A direct repeat sequence of 13 bp (TTCCC[g/a/c]AC[a/g]ATGA) with 11 sites conserved was identified; variations in the 2 variable sites does not correlate with the phylogeny for the ICE (Fig. 3b). The findings from this comparative genomic analysis are consistent with previous smaller comparisons [11, 16, 17], and collectively provide strong evidence for a common mobile element for the *vanD* locus. Phylogenetic trees constructed for both the *vanD* locus (Fig. 3a) and the ICE sequences (Fig. 3b) were highly congruent in their structure, suggesting that both elements are commonly mobilized together. Collectively, these investigations support a hypothesis that the identified ICE is the principal element facilitating mobility of the *vanD* locus and is likely highly specific in its target site for integration.

## Conclusions

Here we present a case study of the *de novo* emergence of *vanD* VREfm during a complex and prolonged infection and add to the understanding of the genomic mechanisms involved. Complete genome reconstruction enabled identification of the *vanD*-harbouring putative ICE. The element does not appear to be autonomously transferable and is stably fixed within the AUS2002 chromosome. This likely represents a stepwise adaptive process in AUS2002, resulting from prolonged antibiotic exposure; acquisition of ICE*VREfm_vanD* into AUS2001 to gain glycopeptide resistance, followed by disruption of the native ddl ligase to enhance the resistance mechanisms and a large chromosomal inversion of unknown impact (the order of the last two events is unclear). While a resistance phenotype was gained, a consequence is that AUS2002 is likely now dependent on the acquired *vanD* ligase for cell wall cross-linking and excision of the ICE may be lethal to the cell. Comparison of the limited number of available *vanD*-harbouring sequences suggested that many *vanD* VRE have evolved through a similar evolutionary pathway to AUS2002. The diverse taxonomic, geographical and host origins for these isolates largely preclude transmission as an explanation for the spread of the *vanD* locus, with *de novo* generation being far more plausible. However, for this to occur the origin species or other carriers of the putative ICE would need to be globally disseminated. In lieu of regular molecular or sequence-based typing, the frequency with which *vanD* VRE emerge is unknown. Further, the circumstances that promote mobilization of the ICE are still unclear, but have important implications for managing the risk of future *vanD* VRE emergence events.

## Supplementary Data

Supplementary material 1
